# The Potential Use of Mitochondrial Extracellular Vesicles as Biomarkers or Therapeutical Tools

**DOI:** 10.3390/ijms24087005

**Published:** 2023-04-10

**Authors:** Jorge Sanz-Ros, Cristina Mas-Bargues, Nekane Romero-García, Javier Huete-Acevedo, Mar Dromant, Consuelo Borrás

**Affiliations:** 1 Freshage Research Group, Department of Physiology, Faculty of Medicine, University of Valencia, Centro de Investigación Biomédica en Red Fragilidad y Envejecimiento Saludable-Instituto de Salud Carlos III (CIBERFES-ISCIII), INCLIVA, 46010 Valencia, Spain; 2 Department of Cardiology, Hospital Universitari i Politècnic La Fe, 46026 Valencia, Spain; 3 Department of Anesthesiology and Surgical Trauma Intensive Care, Hospital Clinic Universitari de Valencia, University of Valencia, 46010 Valencia, Spain

**Keywords:** MitoEVs, mitochondria, extracellular vesicles, biomarker, therapy

## Abstract

The mitochondria play a crucial role in cellular metabolism, reactive oxygen species (ROS) production, and apoptosis. Aberrant mitochondria can cause severe damage to the cells, which have established a tight quality control for the mitochondria. This process avoids the accumulation of damaged mitochondria and can lead to the release of mitochondrial constituents to the extracellular milieu through mitochondrial extracellular vesicles (MitoEVs). These MitoEVs carry mtDNA, rRNA, tRNA, and protein complexes of the respiratory chain, and the largest MitoEVs can even transport whole mitochondria. Macrophages ultimately engulf these MitoEVs to undergo outsourced mitophagy. Recently, it has been reported that MitoEVs can also contain healthy mitochondria, whose function seems to be the rescue of stressed cells by restoring the loss of mitochondrial function. This mitochondrial transfer has opened the field of their use as potential disease biomarkers and therapeutic tools. This review describes this new EVs-mediated transfer of the mitochondria and the current application of MitoEVs in the clinical environment.

## 1. Introduction

The mitochondria are cellular organelles with a double membrane structure that use aerobic respiration to generate ATP. Apart from their traditional role in oxidative phosphorylation, the mitochondria have key roles in several metabolic pathways, cell proliferation, and differentiation, ROS production and consumption, and apoptosis [1]. The mitochondria also have an important role in the fine modulation of calcium homeostasis, and when compromised, they lead to different pathological conditions [2]. Moreover, it has been shown that the alteration of calcium signals that reach the mitochondria during pathological conditions (such as oxidative stress) is accompanied by a deformation of this organelle structure and function, triggering its clearance [3].

Mammal mitochondria contain several copies of their genome consisting of a circular DNA molecule of 16.6 kb. The mitochondrial genome includes 37 genes encoding 13 proteins for subunits of the respiratory complexes of the electron transport chain, 22 tRNA, and 2 rRNA (12S and 16S rRNA).

The mitochondria are transmitted to subsequent generations through the vertical maternal lineage in mammals. During symmetric cell division, the mitochondria are distributed randomly between daughter cells [4]. Conversely, during asymmetric cell division, the mitochondria are differentially segregated. This was demonstrated using a colored labeling strategy where old mitochondria were labeled 48 to 58 h before cell division, and young mitochondria were labeled 0 to 10 h before division. The authors observed that, after cell division, the old-labeled mitochondria from the mother cells were divided more asymmetrically between daughter cells than the young-labeled mitochondria [5], thus suggesting the existence of active mechanisms that guide mitochondria partitioning between daughter cells.

Recently, the mitochondria have been found to be horizontally transferred between mammalian cells, challenging the current concepts of mitochondria inheritance [6]. Diverse structures mediate intercellular mitochondrial transfer. These include tunneling nanotubes (TNTs) [7], extracellular vesicles (EVs) [8], gap junctions [9], and cell fusion [10]. However, free extracellular mitochondria have also been found in supernatants from cells cultured in vitro, as well as in biological fluids, under both physiological and pathological conditions [11,12].

TNTs, gap junctions, and cell fusion have been extensively described; therefore, in this review, we will focus on mitochondria horizontal transfer through EVs. EVs are double lipid layer-surrounded vesicles that are secreted to the extracellular milieu by almost all cell types and they drive intercellular communication. “EVs” is a general term that englobes several subtypes of cell-released membranous structures, including exosomes, microvesicles, apoptotic bodies, and others, regardless of their biogenesis, size, density, and function [13]. These particles can be isolated from the media of cells in culture and from biological fluids, using different procedures, such as serial ultracentrifugation, ultrafiltration, size-exclusion chromatography, immunoaffinity, or microdevices, among others. The size of EVs ranges from 40 nm to 5 μm [14], and their typical cargo includes proteins, lipids, and nucleic acids. These vesicles are known to be involved in physiological and pathological processes, including the removal of unwanted proteins, antigen presentation, genetic exchange, immune response, inflammation, tumor metastasis, and dissemination of pathogens [15]. Recently, intact organelles such as mitochondria have also been detected in EVs; therefore, EVs are believed to participate in intercellular mitochondrial transfer [16,17].

## 2. MitoEVs

EVs have different sizes, ranging from 40–150 nm (small EVs) to 500–5000 nm (large EVs). Smaller EVs mostly contain genetic material, such as mtDNA [18,19,20,21,22], mtRNA [23], and mitochondrial proteins [24,25,26,27,28]. Larger EVs may contain entire polarized mitochondria [8,29,30,31]. As several discrepancies have been described in the literature, mostly due to differences in the EV isolation protocol used, in this review, we englobe all these extracellular vesicles containing either intact mitochondrion or mitochondrion components, as MitoEVs.

MitoEVs transfer enables the incorporation of mitochondria or their components into the endogenous mitochondrial network of recipient cells. Mitochondrial transfer likely occurs under normal and physiological conditions between cells, suggesting a regular exchange of mitochondria that ensures a balanced heteroplasmy [32]. As an example, it has been reported that mesenchymal stem cells (MSCs) package intact mitochondria into MitoEVs, which are transferred to chondrocytes in the absence of direct cell−cell interactions or stimulus [8]. In addition, it has been proposed that mitochondria are transferred during mouse development [33]. Since embryonic development requires cells to rely on aerobic glycolysis to support rapid cell proliferation [34], this mitochondria transfer might play a role in mitochondrial respiration-linked remodeling [35]. However, more research is needed in this field to assess the specific involvement of MitoEVs in the control of mtDNA heterogeneity and tissue homeostasis during normal development.

Interestingly, MitoEVs can contain both healthy or damaged mitochondria, with different physiopathological consequences on target cells. Recent research has provided solid evidence to support that mitochondria are released from cells for transcellular degradation or transferred to other cells as metabolic support or regulatory messengers [36,37,38,39].

## 3. How and Why Do Cells Release MitoEVs?

The intercellular transfer of MitoEVs plays specific roles in different conditions. On the one hand, mitochondria may be released to the extracellular space during developmental processes, inflammatory activation, and in the process of “garbage clearance” of damaged mitochondria [40]. Indeed, MitoEVs are part of the quality control of the mitochondria. When the mitochondria are damaged, cells activate repair mechanisms such as mitochondrial proteostasis, mitochondrial dynamics (fusion/fission), and mitophagy or trans mitophagy [36], that is, sending damaged mitochondria to the surrounding cells (astrocytes or macrophages) to complete the quality control of the mitochondria [41,42].

On the other hand, it is increasingly being reported that MitoEVs released by healthy MSCs promote anti-inflammatory effects and energy metabolism restoration in target cells [43]. It has been suggested that stressed cells send specific signals, such as ROS, leading to the formation of tunneling nanotubes (TNTs) and the shedding of MitoEVs by healthy cells [17,44], resulting in the transfer of healthy mitochondria to stressed cells, thereby restoring their functionality and rescuing them from apoptosis [6]. This was observed in corneal epithelial cells subjected to oxidative stress, which sent environmental cues to MSC, which responded by releasing MitoEVs, that, once internalized, epithelial cells displayed an enhanced survival capacity, elevated mitochondrial respiration, and a wound healing capacity [45]. Similarly, in patients with myoclonus epilepsy with ragged-red fibers (a mitochondrial disease), MSCs donate MitoEVs to rescue injured cells by improving their aerobic respiration, suppressing apoptosis, and decreasing oxidative stress [46].

### 3.1. MitoEVs for Mitochondria Quality Control

**Figure 1:**
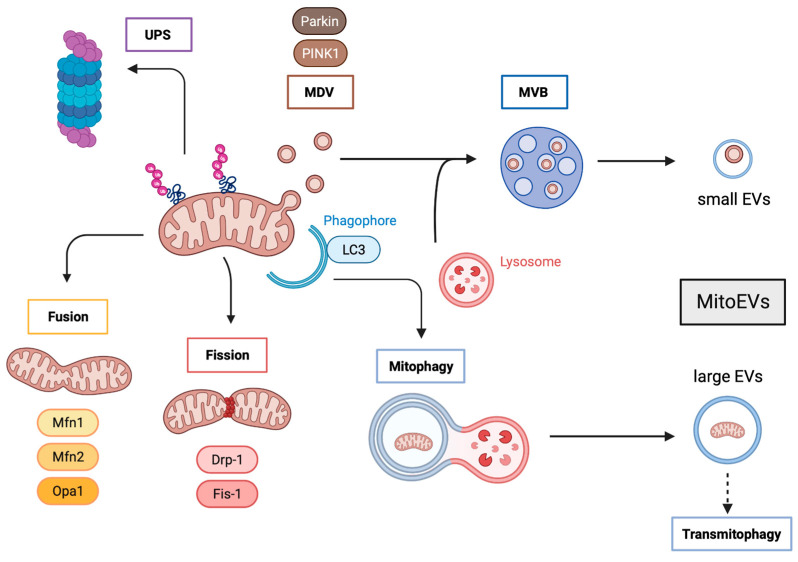
Mitochondria quality control and MitoEVs formation. Aberrant mitochondrial components can be degraded by the UPS, but also be incorporated in MDV that fuse with lysosomes to form MVB, leading to the formation and delivery of small mitoEVs. Whole damaged mitochondria are subjected to fusion and fission processes, the latter ending in mitophagy and promoting transmitophagy through the formation and release of large mitoEVs. UPS: Ubiquitin proteasome system; MDV: mitochondria-derived vesicles; MVBs: multivesicular bodies; PINK1: PTEN-induced putative protein kinase 1; MVB: multivesicular bodies; EVs: extracellular vesicles; Mfn1: mitofusin 1; Mfn2: mitofusin 2; Opa1: optic atrophy 1; Drp-1: dynamin-related protein 1; FIS1: mitochondrial fission protein 1; LC3: microtubule-associated protein 1 light chain 3.

Cells perform mitochondrial quality control through four different pathways (see Figure 1).

The proteostasis of mitochondrial proteins includes mitochondria-localized chaperones and proteases that re-fold or degrade individual aberrant proteins, thereby maintaining the quality of proteins functioning within the mitochondria [47]. Mitochondrial proteostasis degrades unfolded or oxidized proteins within the mitochondrial matrix by mitochondrial proteases; although, in some cases, these aberrant proteins can be ubiquitinated and delivered to the cytosolic ubiquitin−proteasome system (UPS) [48,49,50]. It has been reported that ubiquitination occurs at the inner mitochondrial membrane and that some metabolic proteins (such as succinate dehydrogenase subunit A) are UPS-dependent [51]; thereby suggesting that UPS is involved in the regulation of mitochondrial quality control.

Mitochondrial dynamics consist of the antagonistic and balanced activities of the fusion and fission machinery to shape the mitochondrial compartment. A shift toward fusion favors the generation of interconnected mitochondria, whereas a shift toward fission produces numerous mitochondrial fragments [52]. Large mitochondrial networks generated by fusion are typically observed in metabolically active cells; in contrast, in quiescent cells, the mitochondria are frequently observed as numerous small spheres or short rods. Mitochondrial dynamics are mediated by fusion factors (mitofusin 1 and 2 (MFN1 and MFN2), and optic atrophy 1 (OPA1)) and fission factors (dynamin-related protein 1 (DRP1) and mitochondrial fission protein 1 (FIS1)). Fusion dilutes damaged mitochondria along the network, whereas fission targets dysfunctional mitochondria to their subsequent clearance through mitophagy [53,54]. Depending on the physiological context, MFN2 can either mediate mitochondrial fusion or recruit cytosolic Parkin to initiate mitophagy [55]. Interestingly, alterations in MFN2 can hamper mitochondrial fusion leading to the formation of clumped mitochondrial aggregates [56]. It is likely that in this scenario, clumped mitochondria would also be subjected to degradation through mitophagy.

Severely damaged mitochondria are incorporated into LC3-positive autophagosomes that will eventually fuse with lysosomes or late endosomes for their degradation through mitophagy [57]. This pathway relays on PTEN-induced putative protein kinase 1 (PINK1) and Parkin, which are activated following a loss of mitochondrial membrane potential [58]. Interestingly, it has been reported that autophagy-deficient cells [59,60,61] as well as UPS-deficient cells release an increased number of MitoEVs. Similarly, several stresses increase the number of released MitoEVs. Indeed, under cold stress, brown adipocytes eject MitoEVs containing oxidatively-damaged mitochondria that are cleared by resident macrophages [26,62]. In addition, mesenchymal stem cells (MSC) subjected to oxidative stress package mitochondria into EVs for cellular transfer, which are posteriorly engulfed by macrophages that undergo outsource mitophagy [63].

Mildly damaged mitochondria, not yet completely depolarized, may be also subjected to PINK1 and Parkin action to generate mitochondria-derived vesicles (MDVs) [64,65]. MDVs are generated through the selective incorporation of mitochondrial proteins. These MDVs have a relatively uniform size, between 70 and 150 nm [66]. MDVs have two different fates: they can either fuse with peroxisomes or merge with the endolysosomal system, forming multivesicular bodies (MVBs) that will in turn be released into the extracellular compartment as MitoEVs [67,68]. It is believed that mitochondrial discharge by MitoEVs operates as a first line of defense against partially depolarized mitochondria, before complete depolarization. Moreover, under stress conditions, lysosomal degradation might be exceeded and the MDV containing dysfunctional parts of the damaged mitochondria could then accumulate and act as pro-inflammatory damage-associated molecular patterns (DAMPs) [28,69]. Cells will prevent this chaos by packaging MDVs into multivesicular bodies to be extracellularly discharged as MitoEVs, which are degraded by surrounding macrophages. Very recently, it has been suggested that the elimination of damaged mitochondria via MitoEVs is increased when the lysosomal function is compromised [70], and this mechanism seems to be mediated by the small GTPase Rab7 [71].

In response to stress, it has been published that adipocytes release MitoEVs originating from MDV, which include damaged mitochondria. These MitoEVs are taken up by cardiomyocytes, where they trigger a burst of ROS creating oxidative stress; this results in compensatory antioxidant signaling activation consistent with a metabolic pre-conditioning of the heart [26].

### 3.2. MitoEVs for Rescuing Damaged Cells

Accumulating shreds of evidence suggest that MSCs play a role in the protection of the surrounding damaged cells by providing their intact mitochondria via MitoEVs. It has been reported that mitochondrial transfer through MitoEVs can rescue stressed cells by restoring the loss of mitochondrial function in recipient cells and increasing their metabolic activity [16,24,72]. In a model of acute respiratory distress syndrome (ARDS), it was shown that MSC-released MitoEVs could restore barrier integrity and normal levels of oxidative phosphorylation, thereby reducing lung injury [73].

Mitochondrial function is involved in maintaining and dictating stem cell fate, which plays a role in metabolic reprogramming during quiescence, activation, self-renewal, proliferation, and differentiation. As the mitochondria produce most of the energy by oxidative phosphorylation, the switch of energy supply from glycolysis to aerobic metabolism is essential for the successful differentiation or reprogramming of recipient cells. Indeed, the transfer of healthy mitochondria can reprogram the differentiated cells [74]. It has been reported that platelets also shed MitoEVs that are integrated by MSCs, activating their pro-angiogenic activity via their metabolic remodeling [75], suggesting that MitoEVs promote tissue repair processes.

The mitochondria have a key role in immune-cell regulation. The mitochondria promote ROS signaling and metabolite availability within immune cells and act as a scaffold for protein interaction. Therefore, the mitochondria are believed to be necessary for immune cells to fulfill their specific role in both innate and adaptive responses [76]. MitoEVs can be integrated by T cells and alter their mitotic processes [29]. Recent studies have shown that macrophages uptake MitoEVs released by MSCs, which stimulates their mitochondrial activity [77]. A study reported that MitoEVs released by healthy MSCs ameliorated acute lung injury because macrophages that engulfed these MitoEVs had enhanced phagocytic capacity and reduced the secretion of TNFα, thereby suppressing lung inflammation [17]. Importantly, macrophages that have engulfed MitoEVs might display either pro- or anti-inflammatory effects. It seems that the pro-inflammatory effects have been described when MitoEVs are released by MSC subjected to pro-inflammatory treatments such as LPS, whereas the anti-inflammatory effects were observed in resting MSC [77,78]. These findings suggest that MitoEVs regulate the immune system.

Interestingly, it has recently been reported that macrophages accumulate in peripheral nervous tissue and donate their mitochondria through EVs to sensory neurons to support pain resolution [79]. This discovery opens a novel set of strategies to resolve chronic pain through the restoration of mitochondrial homeostasis in neurons or by enhancing the transfer of the mitochondria from the macrophages. Moreover, it has been reported that in a model of cerebral ischemia, astrocytes release MitoEVs to protect neurons from hypoxia and glucose deprivation [80].

The following sections are dedicated to review the current use of MitoEVs as therapeutic tools, as well as their role as biomarkers for disease diagnosis.

## 4. Potential Use of MitoEVs as Diagnostic Markers

Similar to other subtypes of EVs, MitoEVs are altered in several diseases, including cancer, neurodegenerative disorders, and cardiovascular disease [69]. MitoEVs contain a variety of molecular components from releasing cells, including proteins, lipids, and nucleic acids, which may serve as indicators of disease status [69]. Here, we discuss how differences in the content and markers of these vesicles could thus be used as diagnostic tools for distinct conditions.

### 4.1. Cancer

The search for new methods to diagnose cancer in its early stages and distinguish between states of the disease has led to the development of liquid biopsies. The analysis of body fluids such as blood or urine to gather information about a person’s cancer status has emerged as a powerful tool for cancer diagnosis, prognosis, and treatment monitoring, as it allows for the detection of cancer-related genetic alterations in a minimally invasive manner [81,82].

Cancer cells often release various types of molecules into the bloodstream, including DNA, RNA, and proteins [83]. These molecules can be used to detect cancer cells and track their progression over time. The results of a liquid biopsy can provide important information about the type and stage of cancer, as well as help monitor the effectiveness of the treatment and detect the early signs of cancer recurrence [82].

The analysis of EVs in liquid biopsies has emerged as a novel method to provide new insights into the role of EVs in several diseases, as the content in EVs varies across disease status [84]. Currently, the main application of the analysis of EVs in liquid biopsies is in the detection and characterization of cancer-specific biomarkers [84,85]. This approach offers several advantages over traditional diagnostic methods, such as tissue biopsy or imaging. Firstly, EVs are readily available in the bloodstream, making liquid biopsy with EVs a minimally invasive and convenient option for cancer diagnosis and monitoring. Secondly, EVs contain a wealth of information about cancer cells, including their genetic and epigenetic alterations, which can provide valuable insights into cancer’s biology, progression, and treatment response [86].

The mitochondria, the cellular organelles responsible for energy production, have emerged as crucial players in the development and progression of cancer. Growing evidence links mitochondrial dysfunction to various aspects of cancer biology, including metabolism, apoptosis, and signaling pathways [87]. In this context, it has been shown that cancer cells release EVs that contain specific mitochondria-derived molecules, such as proteins or mtDNA [18,88,89].

mtDNA present in EVs has an important role in cancer biology and progression, making it an interesting source in cancer diagnosis. mtDNA transfer between cancer cells acts as an oncogenic signal, promoting the escape of cells from metabolic quiescence [20]. Similarly, mtDNA contained in metastatic tumor cells is transferred to low-metastatic tumor cells via MitoEVs, enhancing the metastatic potential during tumor progression [22]. In a more recent study, the authors showed that the protein PINK1 mediates the packaging of mtDNA in EVs from cancer cells and that this mtDNA can promote invasiveness through the activation of Toll-like receptor 9 in recipient cells [21].

Some studies have proposed that MitoEVs could serve as new biomarkers of cancer. Jang et al. discovered that EVs released by melanoma tissue contain higher levels of mitochondrial membrane proteins when compared with non-cancerous cells. In addition, they found that patients with melanoma or other types of cancer such as ovarian or breast cancer have a higher concentration in the plasma of these MitoEVs [25]. Regarding mtDNA, it was recently shown that patients with pancreatic ductal adenocarcinoma have a higher enrichment of mtDNA in circulating EVs, detecting specific mtDNA mutations that could serve as a tool for early cancer detection [90]. Moreover, mtDNA contained in MitoEVs obtained from the plasma exhibit different characteristics among patients with hepatocellular carcinoma, hepatitis, or healthy individuals, indicating a potential role as a diagnostic biomarker in these conditions [91].

### 4.2. Other Diseases

Although cancer is currently the most studied disease in terms of liquid biopsies and MitoEVs, recent studies have found that the content in MitoEVs can be altered in other diseases, such as neurological or cardiovascular conditions [69].

Multiple lines of evidence suggest that mitochondrial dysfunction plays a key role in the pathogenesis of Parkinson’s disease (PD). Post-mortem studies have shown that there is a reduction in the number and size of mitochondria in the substantia nigra region of PD patients’ brains [92]. Additionally, there is evidence of decreased mitochondrial respiratory chain activity and increased ROS generation in PD patients [93]. Furthermore, mutations in genes that regulate mitochondrial function, such as PINK1 and Parkin, are associated with some forms of PD [94]. Recently, it was shown that these proteins are involved in mitochondrial quality control through the regulation of mitochondria-derived vesicle trafficking [64,95]. Along with these results, a clinical study with PD patients suggested that circulating EVs were altered in the disease; more specifically, they found that a specific mitochondrial signature was present in these patients [96].

Another neurological condition characterized by mitochondrial dysfunction is Down syndrome (DS). Patients have impairments in mitochondrial function, which leads to a decrease in energy production that may contribute to the cognitive impairments seen in individuals with Down syndrome [97]. Additionally, studies have shown that people with Down syndrome have an increased susceptibility to oxidative stress [98]. A recent study that presents a new approach to isolate and separate EV subpopulations from the brain extracellular matrix, identifies a unique subset of EVs of a mitochondrial origin, which they term mitovesicles. The authors found that the number and composition of brain mitovesicles are altered in individuals with DS, indicating their possible role in the neuropathological process [27].

In cardiovascular disease, mitochondrial dysfunction has been linked to the development of key pathological changes such as heart failure or atherosclerosis [99,100]. MitoEVs regulate mitochondrial quality control in the cardiovascular system [101,102] and serve as pro-inflammatory signaling between monocytes and endothelial cells in cardiovascular disease [103]. This particular subtype of vesicles has a crucial role in the maintenance of mitochondrial homeostasis in the heart, as cardiomyocytes release dysfunctional mitochondria taken up by resident macrophages [104].

**Figure 2:**
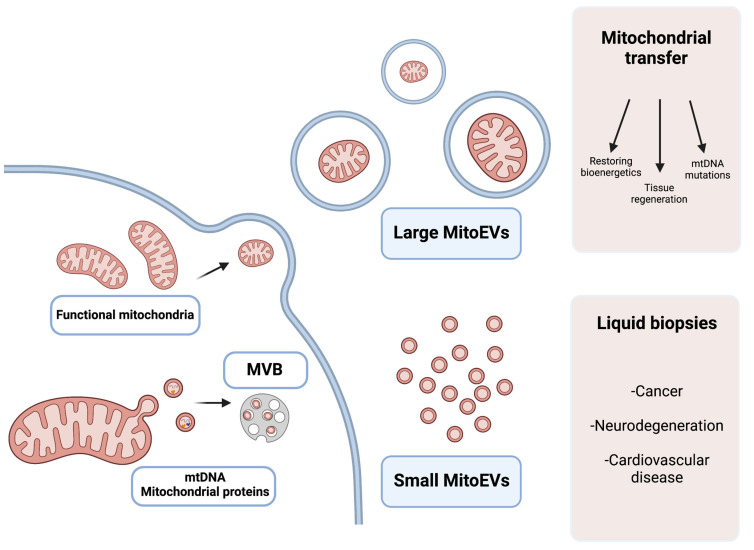
Graphical explanation of the potential therapeutic and diagnostic use of large and small MitoEVs. Larger MitoEVs refer to EVs that are shed from the plasmatic membrane of the cell and can include whole mitochondria; these EVs are particularly interesting in the field of mitochondrial transfer as therapeutic vehicles. Small MitoEVs are included in MVB previous to their release and contain material from the mitochondrial origin (mtDNA and proteins); its analysis may serve as a diagnostic tool in liquid biopsies.

Thereby, MitoEVs can be detected in biological fluids and seem to play a role in the regulation of mitochondrial biology and intercellular communication, making them an interesting subtype of EVs that could be used as diagnostic markers for several diseases (Figure 2 and Table 1).

## 5. MitoEVs as Therapeutic Tools

EV therapy has shown potential in a variety of applications, including regenerative medicine, cancer treatment, and immune modulation. They play a crucial role in intercellular communication and have been recognized as potential therapeutic agents. One of the major advantages of extracellular vesicle therapy is that it avoids some of the limitations associated with traditional cell-based therapies. EVs have a lower risk of immunogenicity compared with cells, and greater stability and longer half-lives compared with other biological therapeutics [118,119,120,121,122].

As a fairly novel field, the use of MitoEVs as a therapy is not well established; the main challenge that this approach faces is that classic isolation methods for EVs mainly distinguish subtypes of these vesicles by size [121], making it difficult to separate a specific subset that comes from a mitochondrial origin. Currently, the main focus of MitoEVs as therapeutic agents is in the field of mitochondrial transfer (Figure 2 and Table 1). Mitochondrial transfer is a therapeutic strategy that involves transferring healthy mitochondria to cells with dysfunctional mitochondria [123,124]. Mitochondrial transfer offers a promising avenue for the treatment of several diseases, as it addresses the root cause of mitochondrial dysfunction. There are several approaches to mitochondrial transfer, including microinjection, the fusion of cells, and the use of EVs as transfer vehicles [124]. The latter strategy seems to be the more feasible, as EVs are already being studied as therapeutics, and may serve as mitochondria transfer vehicles in a non-invasive manner.

The mitochondrial transfer has been used in preclinical models of several diseases. One of the most studied settings is the use of these healthy mitochondria to improve tissue regeneration. During tissue regeneration, cells must undergo a series of complex processes, including cell proliferation, differentiation, and migration. These processes require high levels of energy, and the mitochondria play a crucial role in providing this energy [125]. They also play a crucial role in regulating cell signaling during tissue regeneration. They are involved in the production of signaling molecules, such as reactive oxygen species, which can activate the signaling pathways that regulate cell proliferation and differentiation [123,126]. The modulation of mitochondrial calcium trafficking has also been highlighted as a potential target in tissue regeneration and other pathophysiological contexts [2]. In addition, studies have shown that mitochondrial dysfunction is a key factor that leads to impaired tissue regeneration in aging [127].

The transfer of healthy mitochondria has been successfully used to improve tissue regeneration in models of myocardial ischemia and reperfusion injury (IRI) [105], brain ischemia [106,107], limb ischemia [109], lung IRI [110], and acute kidney failure [111]. In brain ischemia, transplanted mitochondria are incorporated into various cells, resulting in increased ATP content, complex IV expression, and neurogenesis, while also reducing oxidative stress, apoptosis, and inflammatory responses [106,107,108,128]. In cardiac and limb ischemia, transplanted mitochondria enhance ATP production and synthesis, improve cell viability, and activate proteomic pathways for energy production, mitochondrial function, and cellular respiration, while reducing pro-inflammatory markers and inhibiting endoplasmic reticulum stress and caspase-3 expression [105,109,129]. Further research is needed to determine the effects of these mitochondria on other tissues and damaging agents. Interestingly, researchers recently identified a stress response in adipocytes that prevents oxidative damage in the heart through the release of EVs with mitochondrial content [26]. In the same line, mitochondria-rich EVs from autologous cardiomyocytes derived from stem cells have been shown to improve the bioenergetics of the ischemic heart [31], as well as an increment of the viability of cardiomyocytes in a model of doxorubicin injury [112].

It is interesting to note that many of these pathways affected by the treatment with a mitochondrial transfer, such as decreasing the inflammatory response, lowering oxidative stress, or regulating apoptosis, are also affected by treatment with EVs in models of tissue damage and regeneration [130], suggesting that there may be common mechanisms of action between these two approaches.

Moreover, as stated earlier, neurodegenerative diseases are characterized by a sharp increase in dysfunctional mitochondria. The transfer of healthy mitochondria has demonstrated positive effects in mouse models of Alzheimer’s and Parkinson’s disease [113,114]. These effects include decreases in neuronal loss, reduced gliosis in the hippocampus, and the amelioration of mitochondrial dysfunction in the brain.

One of the most promising domains of mitochondrial transfer may have a curative role in the field of mitochondrial diseases. Mitochondrial diseases are caused by mutations in mtDNA and affect the function of mitochondria and oxidative phosphorylation. These mutations can lead to a wide range of clinical manifestations, including muscle weakness, neurological symptoms, developmental delay, and organ failure. The severity and pattern of symptoms can vary widely depending on the nature and location of the mtDNA mutation and the level of heteroplasmy [131]. The transfer of healthy mitochondria to cells that have defective mitochondria with mtDNA mutations could help to restore cellular function, as small changes in the ranges of heteroplasmy could lead to improved tissue function [132]. This approach has shown beneficial effects in genetic diseases related to mutations in mtDNA, improving bioenergetics in cells that carry mutated mitochondria [115,116,117].

## 6. Conclusions and Future Perspectives

MitoEVs carry a diversity of mitochondria and mitochondrial components (mtDNA, mtRNA, rRNA, tRNA, and protein complexes). This cargo can be a part of mitochondria quality control, where cells release the trash to the extracellular space. However, this cargo can also be a rescue package for damaged cells uptaking these MitoEVs. Therefore, the exact function of MitoEVs depends on the context of the donor and target cells. To date, the exact mechanism for the selective package of mitochondrial components within MitoEVs is still in its infancy, and more research in the field is needed.

MitoEVs carrying damaged mitochondria components are currently being investigated for their usefulness as early disease biomarkers. There has been a particular emphasis on cancer, neurodegenerative disorders, and cardiovascular diseases regarding diagnosis, prognosis, and treatment monitoring. Currently, the main use of MitoEVs as therapeutic agents is mitochondrial transfer, which involves transferring healthy mitochondria to cells with dysfunctional mitochondria, restoring their energetic profile, and improving the tissue’s regenerative potential.

These findings provide a future pathway for MitoEVs-based therapies and the use of this subtype of EVs as biomarkers. Moreover, the ability of MitoEVs to modulate important pathways and processes, such as immune response, has been highlighted. However, there are still many questions that need to be addressed before expanding the use of MitoEVs is expanded, such as how to separate vesicles from the mitochondrial origin and other subtypes of EVs.

## Figures and Tables

**Table 1:** Summary of applications of MitoEVs in human diseases, regarding the sources of the vesicles and the key findings in different studies.

Application	Disease	Source	Findings	References
Diagnosis	Melanoma, ovarian and breast cancer	Plasma	Mitochondrial protein enriched EVs from cancerous cells are present at higher concentrations in patients’ plasma	[25]
	Pancreatic ductal adenocarcinoma	Plasma	EVs from mitochondria carrying specific mtDNA mutations from cancer cells are present can be detected in patients’ plasma	[90]
	Hepatocellular carcinoma and hepatitis	Plasma	mtDNA profile in plasma MitoEVs differs between patients with hepatocellular carcinoma, hepatitis, and healthy individuals	[91]
	Parkinson’s disease	Plasma	Circulating EVs from PD patients have a specific mitochondrial signature	[96]
Therapy	Myocardial ischemia-reperfusion injury	Healthy cells	Mitochondrial transfer improved tissue regenerative capacity, enhanced ATP production, improved cell viability, and reduced pro-inflammatory markers	[105]
	Brain ischemia	Xenogenic and muscle mitochondria, MSCs	Mitochondrial transfer improved neurogenesis, and reduced pro-inflammatory markers, oxidative stress and apoptosis	[106,107,108]
	Limb ischemia	Healthy cells	Mitochondrial transplantation improved tissue regenerative capacity, enhanced ATP production, improved cell viability, and reduced pro-inflammatory markers	[109]
	Lung ischemia-reperfusion injury	Healthy cells	Mitochondrial transplantation improved tissue regenerative capacity	[110]
	Acute kidney injury	Healthy cells	Intra-arterial mitochondrial transplantation improved tissue regenerative capacity	[111]
	Doxorubicin injury to cardiomyocytes	MSCs	Mitochondria-rich EVs improve cell viability in induced cardiomyocytes from patients with doxorubicin injury	[112]
	Alzheimer’s disease	Healthy cells	Mitochondrial transfer improves cognition and lower neuronal loss and gliosis in mice	[113]
	Parkinson’s disease	Allogenic and xenogenic mitochondria	Mitochondrial transplantation restored mitochondrial function and attenuated 6-hydroxydopamine-induced neurotoxicity in mice	[114]
	Mitochondrial diseases	Healthy cells	Mitochondrial transfer can improve mitochondrial bioenergetics in cells from patients with mutations in mtDNA	[46,115,116,117]

## Data Availability

Data sharing not applicable.
